# Orthostatic Blood Pressure Test for Risk Stratification in Patients with Hypertrophic Cardiomyopathy

**DOI:** 10.1371/journal.pone.0131044

**Published:** 2015-06-24

**Authors:** Julia Münch, Ali Aydin, Anna Suling, Christian Voigt, Stefan Blankenberg, Monica Patten

**Affiliations:** 1 Klinik und Poliklinik für Allgemeine und Interventionelle Kardiologie, Universitäres Herzzentrum Hamburg, Martinistr. 52, 20246, Hamburg, Germany; 2 Krankenhaus Reinbek, Abteilung für Kardiologie, Hamburger Strasse 41, 21465, Reinbek, Germany; 3 Institut für Medizinische Biometrie und Epidemiologie, Universitätsklinikum Hamburg Eppendorf, Martinistr. 52, 20246, Hamburg, Germany; KRH Robert Koch Klinikum Gehrden, GERMANY

## Abstract

**Background:**

Hypertrophic cardiomyopathy (HCM) is the most common cause of sudden cardiac death (SCD) in young adults, mainly ascribed to ventricular tachycardia (VT). Assuming that VT is the major cause of (pre-) syncope in HCM patients, its occurrence is essential for SCD risk stratification and primarily preventive ICD-implantation. However, evidence of VT during syncope is often missing. As the differentiation of potential lethal causes for syncope such as VT from more harmless reasons is crucial, HCM patients were screened for orthostatic dysregulation by using a simple orthostatic blood pressure test.

**Methods:**

Over 15 months (IQR [9;20]) 100 HCM patients (55.8±16.2 yrs, 61% male) were evaluated for (pre-)syncope and VT (24h-ECGs, device-memories) within the last five years. Eighty patients underwent an orthostatic blood pressure test. Logistic regression models were used for statistical analysis.

**Results:**

In older patients (>40 yrs) a positive orthostatic test result increased the chance of (pre-) syncope by a factor of 63 (95%-CI [8.8; 447.9], p<0.001; 93% sensitivity, 95%-CI [76; 99]; 74% specificity, 95%-CI [58; 86]). No correlation with VT was shown. A prolonged QTc interval also increased the chance of (pre-) syncope by a factor of 6.6 (95%-CI [2.0; 21.7]; p=0.002).

**Conclusions:**

The orthostatic blood pressure test is highly valuable for evaluation of syncope and presyncope especially in older HCM patients, suggesting that orthostatic syncope might be more relevant than previously assumed. Considering the high complication rates due to ICD therapies, this test may provide useful information for the evaluation of syncope in individual risk stratification and may help to prevent unnecessary device implantations, especially in older HCM patients.

## Introduction

Hypertrophic cardiomyopathy (HCM) is the most common monogenic inherited cardiovascular disease and the most frequent cause of sudden cardiac death (SCD) in young athletes, while being a less common cause in the elderly [1]. As syncope is a common manifestation in all mutation carriers independent of their clinical phenotype, identification of patients at high risk of SCD is essential regarding the clinical decision for prophylactic ICD implantation [2]. Six major clinical parameters for risk stratification are recommended, such as otherwise unexplained syncope, previous cardiac arrest, sustained or non-sustained ventricular tachycardia (VT), extreme left ventricular hypertrophy, abnormal blood pressure response to exercise, or a family history of SCD. However, the latest up-date of international guideline recommendations for ICD implantation in HCM patients presents a new risk score in which age plays a more prominent role in risk stratification based on the observation that VT in older patients fails to predict SCD [3]. While malignant arrhythmias are assumed to be a relevant cause of syncope in HCM, retrospective assessment of these episodes is limited, as documentation of an underlying arrhythmia is usually missing. In elderly patients, syncope is rather common, whereas the incidence of HCM related SCD decreases over lifetime. Consequently, there is a need for more valid clinical parameters in individual risk stratification, especially to uncover the etiology of syncope in older HCM patients.

The orthostatic blood pressure test is part of cardiologic routine in evaluating otherwise unexplained vertigo and syncope in non-HCM patients and is proven to be useful for the diagnosis of orthostatic dysregulation [2]. However, its impact in evaluating syncope and presyncope in HCM patients is unclear.

In this regard, a single centre observational study was performed to analyse the relationship between orthostatic dysregulation and the occurrence of cardiac adverse events, such as syncope, presyncope and VT in HCM patients. In addition, QTc prolongation as an independent risk factor for VT was also assessed.

## Materials and Methods

### Study population

One hundred HCM patients (≥ 18 yrs) were recruited between January 2011 and June 2013 and observed for a median duration of 15 months (IQR [9; 20]) in our outpatient clinic. Diagnosis of HCM was based on two-dimensional echocardiographic evidence of a hypertrophied, non-dilated left ventricle with maximum wall thickness of ≥ 15 mm, in the absence of abnormal loading conditions or another cardiac or systemic disease that could produce the magnitude of hypertrophy evident [3–5]. All patients with additional hypertension were diagnosed of HCM according to at least one of the following criteria: hypertension occurring years after the diagnosis of HCM, detection of HCM-causing gene mutation or family history of HCM, maximum wall thickness exceeding the expected dimension caused by hypertension alone (i.e. ≥ 20 mm), presence of marked mitral leaflet elongation [6], dynamic LVOT obstruction (≥ 30 mmHg) at rest, and distribution of late gadolinium enhancement (LGE) by cardiac magnetic resonance imaging (CMR) consistent with HCM [3,7,8]. Previous data suggest that an abnormal blood pressure response to physical exercise is only reliable for the prediction of SCD in patients younger than 40 years of age [9]. We therefore focused on patients ≥ 40 years in a subgroup analysis.

### Ethics statement

The study protocol was in line with the principles outlined in the Declaration of Helsinki. The research protocol was approved by the local ethics committee of the “Ärztekammer Hamburg”, Germany, and all subjects gave written informed consent to participate in the study.

### Assessment of adverse events

Adverse events were defined as the occurrence of not otherwise explained syncope and presyncope, or documented VT. Twice a year each patient was interviewed for syncope and presyncope during their routine visits. In 35 patients with implanted devices, the device’s memory was read out to detect VT. In patients without a device, an ambulatory 24h-ECG was performed at least once a year. A non-sustained VT was defined as a ventricular run of more than three action potentials [3]. All reported or documented events within the last five years before enrolment were assessed retrospectively.

### ECG and assessment of the QTc interval

A 12-lead electrocardiogram (ECG) was routinely performed at each visit. The QTc interval represents the duration of cardiac depolarisation and subsequent repolarisation adjusted to heart frequency [10]. Automatic measurements were used, which other studies have proved to be valid and reproducible [11]. All leads were recorded simultaneously, providing great accuracy in distinguishing the different phases of de- and repolarisation, and automatically measured QT values were visually validated for plausibility [10]. QTc intervals of 450 ms (men) and 470 ms (women) were taken as cut-off points.

### Echocardiography

Two-dimensional transthoracic echocardiography was performed using an E133 Philips ultrasound system, assessing LV ejection fraction and maximum end-diastolic wall thickness. LVOT obstruction was identified by a peak instantaneous outflow gradient ≥30 mmHg at rest or ≥50 mmHg under valsalva maneuver or symptom limited exercise.

### Laboratory values

NT-proBNP values and routine laboratory tests were taken, comprising blood count, creatinin, transaminases, C-reactive protein (CRP) and thyroid-stimulating hormone (TSH) to rule out other important causes for orthostatic hypotension, like renal impairment, anemia, infections or hypothyroidism.

### Orthostatic blood pressure test

Eighty patients were investigated using the orthostatic blood pressure test. Heart rate and blood pressure were measured every minute using a DINAMAP device by applying oscillometry, stepwise deflation and a two-tube inflate/monitor system to gain the best possible accuracy in blood pressure measurements. Values were initially obtained with the patient lying horizontally for five minutes and subsequently in upright position for further ten minutes. Orthostatic hypotension was defined as a decrease of at least 20 mmHg in systolic- and/or 10 mmHg in diastolic blood pressure within the first three minutes after changing from supine to upright position. All values were obtained during daily routine in our outpatient clinic and the respective nurse was blinded to the underlying disease of the patient.

### Statistical analysis

Continuous variables are reported as mean ± standard deviation (SD) or interquartile ranges (IQR) and categorical variables are presented as frequencies and percentages. Logistic regressions were used to evaluate possible influence of medication, NT-proBNP value, septum wall thickness and the LVOT gradient on the orthostatic test and a prolonged QTc interval. To determine the performance of the orthostatic test and a prolonged QTc interval as a predictor for adverse events, we calculated sensitivity, specificity, positive predictive values (PPV) and negative predictive values (NPV) with 95% confidence intervals (CI) and logistic regressions. All models were adjusted for age and sex. Odds ratios (OR) with 95%-CI are presented, as well as adjusted prevalence where appropriate. A two-tailed p-value <0.05 was considered statistically significant. All analyses were carried out using STATA 13 (StataCorp. 2013).

## Results

Baseline characteristics are listed in Table 1. Of all patients, 25% showed LVOT obstruction ≥30 mmHg at rest and additional 32% had a significant LVOT obstruction ≥50 mmHg during exercise. Of all patients 24 were treated for arterial hypertension and 17 patients had paroxysmal atrial fibrillation.

**Table 1:** Baseline characteristics of HCM patients. Values are given as total number of patients (n) or as mean ± SD.

	patients with ECG (n = 100)	patients with ECG and orthostatic test (n = 80)	patients with ECG and orthostatic test and ≥ 40y (n = 69)
**male, n (%)**	61 (61.0)	50 (62.5)	39 (56.5)
**female, n (%)**	39 (39.0)	30 (37.5)	30 (43.5)
**age (yrs)**	55.8 ± 16.2	56.1 ± 15.1	60.1 ± 12.0
**maximal wall thickness (mm)**	23.2 ± 5.2	23.2 ± 5.5	22.2 ± 4.3
**gradient at rest (mmHg)**	25.7 ± 29.1	23.9 ± 26.6	25.5 ± 27.9
**gradient max. (mmHg)**	47.0 ± 38.0	43.1 ± 34.8	44.7 ± 35.5
**NT-proBNP (ng/ml)**	1913.9 ± 3970.8	1452.2 ± 2037.2	1456.0 ± 2053.6
**device, n (%)**	35 (35.0)	29 (36.3)	25 (36.2)
**coronary artery disease, n (%)**	11 (11.0)	8 (10.0)	8 (11.6)
**cardiac medication, n (%)**	65 (65.0)	50 (62.5)	45 (66.7)
**betablocker, n (%)**	46 (70.8)	36 (72.0)	33 (73.3)
**verapamil, n (%)**	17 (26.2)	13 (26.0)	11 (24.4)
**other, n (%)**	2 (3.1)	1 (2.0)	1 (2.2)

### Impact of disease related factors and medication on the orthostatic test result and QTc interval

The effect of medication, NT-proBNP value, septum-thickness and the LVOT gradient adjusted for the patient’s age and sex were investigated as predictors for the orthostatic test result and QTc interval. As shown in Tables 2 and 3, none of these factors had a significant influence. Besides verapamil or beta blockers, one patient was taking ranolazine and one diltiazem.

**Table 2:** Influence of disease related factors and medication on the orthostatic test result in 69 HCM patients older than or equal to 40 years.

	orthostatic test positive	orthostatic test negative	OR [95%-CI] (p-value)
**n (%)**	36 (52.2)	33 (47.8)	-
**sex (male), n (%)**	18 (46.2)	21 (53.8)	0.7 [0.2;2.0] (0.473)
**age (yrs)**	61.5 ± 12.5	58.7 ± 11.4	1.0 [1.0;1.1] (0.220)
**septum wall (mm)**	21.9 ± 4.1	22.5 ± 4.5	0.9 [0.8;1.0] (0.199)
**gradient at rest (mmHg)**	32.2 ± 33.2	18.3 ± 18.4	1.0 [1.0;1.0] (0.074)
**NT-proBNP (ng/ml)**	1934.8 ± 2410.7	917.3 ± 1409.9	1.0 [1.0;1.0] (0.220)
**no medication, n (%)**	13 (54.2)	11 (45.8)	-
**betablocker, n (%)**	17 (51.5)	16 (48.5)	0.6 [0.2;2.2] (0.462)
**verapamil, n (%)**	6 (54.6)	5 (45.5)	0.8 [0.2;4.1] (0.836)

Values are given as total number of patients (n) or as mean ± SD.

**Table 3:** Influence of disease related factors and medication on the QTc interval in 100 HCM patients.

	QTc prolonged	QTc normal	OR [95%-CI] (p-value)
**n (%)**	26 (26.0)	74 (74.0)	-
**sex (male), n (%)**	15 (24.6)	46 (75.4)	0.9 [0.3;2.6] (0.813)
**age (yrs)**	60.7 ± 14.4	54.1 ± 16.5	1.0 [1.0;1.1] (0.028)
**septum wall (mm)**	23.9 ± 6.4	22.9 ± 4.8	1.1 [1.0;1.2] (0.123)
**gradient at rest (mmHg)**	20.7 ± 28.7	27.4 ± 29.3	1.0 [1.0;1.0] (0.151)
**NT-proBNP (ng/ml)**	2131.9 ± 1983.0	1832.9 ± 4501.0	1.0 [1.0;1.0] (0.563)
**no medication, n (%)**	12 (34.3)	23 (65.7)	-
**betablocker, n (%)**	12 (25.5)	35 (74.5)	0.4 [0.2;1.3] (0.146)
**verapamil, n (%)**	2 (11.8)	15 (88.2)	0.2 [0.0;1.2] (0.081)

Values are given as total number of patients (n) or as mean ± SD.

### Orthostatic test result as a predictor for adverse events in HCM patients

**Fig 1:**
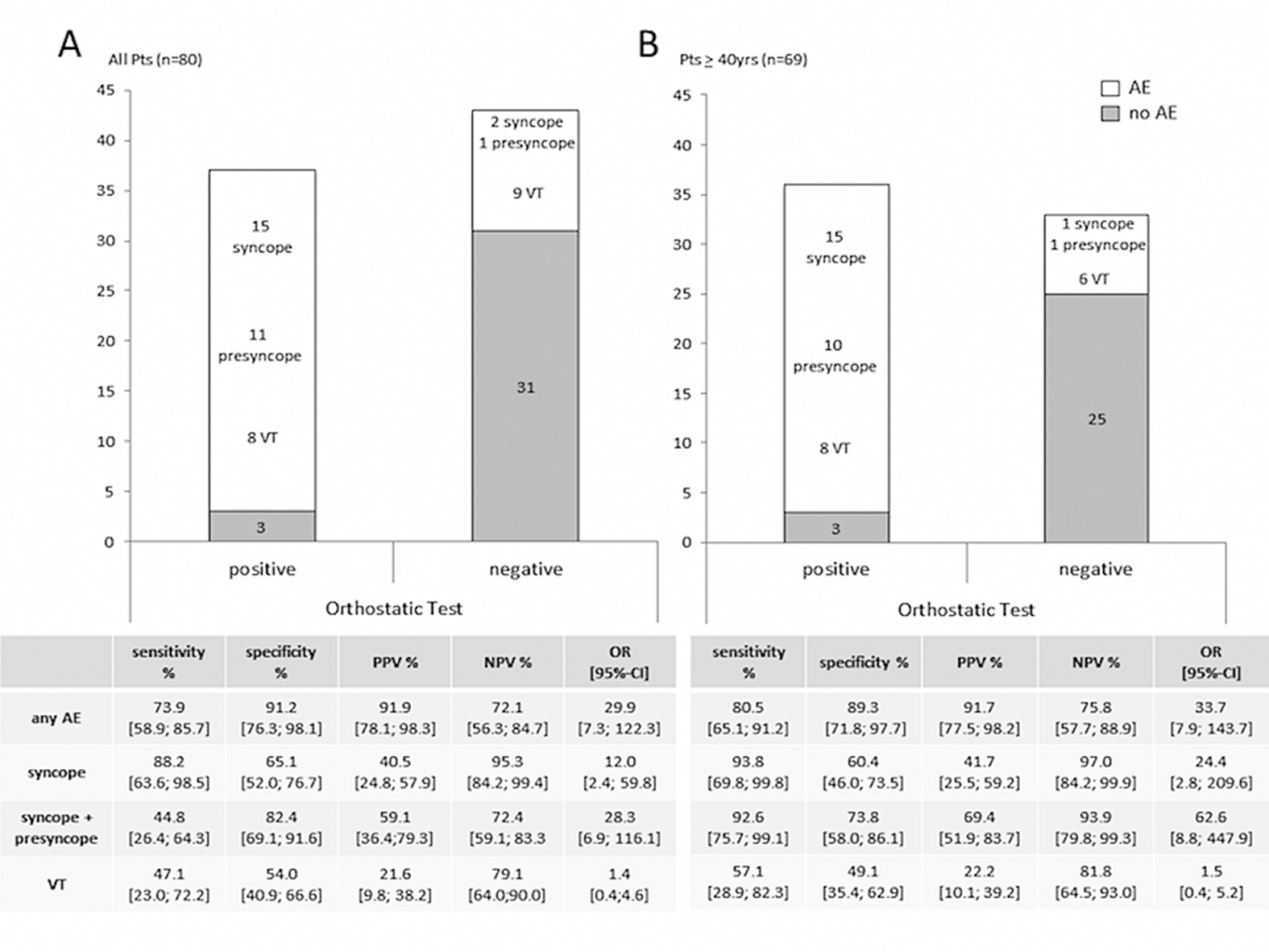
Impact of orthostatic test on the occurrence of adverse events in HCM patients for all patients (A) and for the subgroup of patients > 40 yrs (B). Sensitivity, specificity, PPV (positive predictive value), NPV (negative predictive value) and OR (odds ratio; adjusted for age and sex) with 95%-CI (confidence intervals) are given in the table below. AE = adverse events (syncope, presyncope, VT).

The orthostatic test was positive in 37 (46%) of all patients tested for orthostatic hypotension (Fig 1A) and in 36 (52%) of the patients ≥40 yrs (Fig 1B). Within the last five years before enrollment 46 of 80 patients (58%) had adverse events: 17 (21%) syncope, 12 (15%) presyncope, and 17 (21%) VT. Among the elderly the proportion of adverse events was slightly higher with 59% (41 of 69 patients), splitting up into 16 (23%) syncope, 11 (16%) presyncope and 14 (20%) VT.

Logistic regressions revealed the respective test results to be a significant predictor for the occurrence of adverse events in both groups. Comprising all ages (Fig 1A), the test was highly specific (91%) yet less sensitive (74%), while in the older subgroup (Fig 1B) almost equal specificity (89%) and even higher sensitivity (81%) were observed (positive predictive value (PPV) 92%). In this subgroup, individual analysis of the occurrence of syncope and presyncope taken together revealed an even higher sensitivity (93%) with an almost similar specificity (74%). The negative predictive value (NPV) for the non-occurrence of syncope or presyncope in the presence of a normal test result was 94% and for syncope alone 97% (Fig 1B). Having a positive orthostatic test result increased the chance for syncope by a factor of 24.4 (95-CI [2.8;209.6], p = 0.004) and for syncope or presyncope by a factor of 62.6 (95%-CI [8.8;447.9], p<0.001), compared to individuals with a negative result. However, the prediction for the occurrence of VT was not significant; as VT appeared to be almost homogenously distributed (p = 0.29; Fig 1).

#### QTc interval as a predictor for adverse events

**Fig 2:**
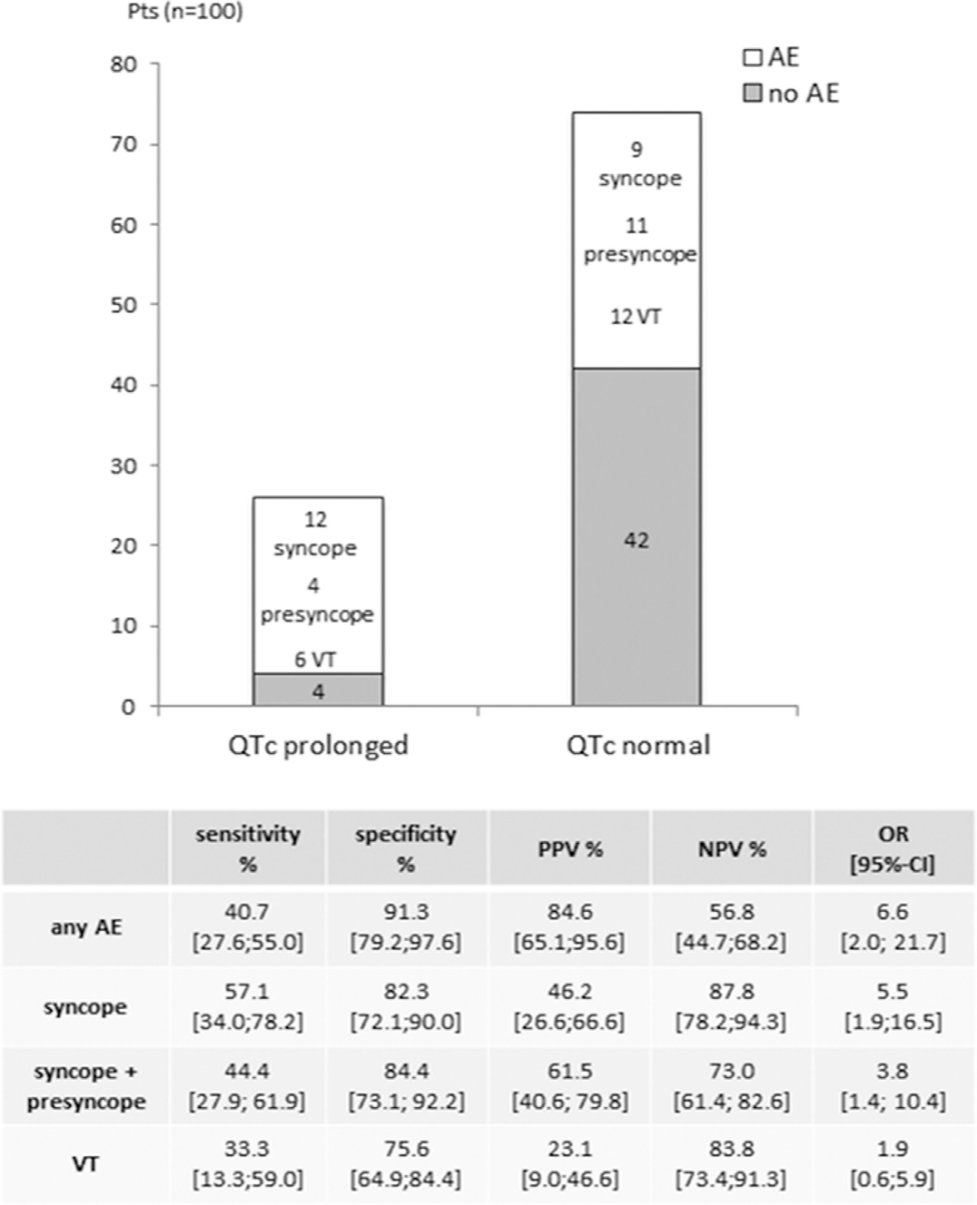
Impact of QTc prolongation on the occurrence of adverse events in HCM patients. Sensitivity, specificity, PPV (positive predictive value), NPV (negative predictive value) and OR (odds ratio; adjusted for age and sex) with 95%-CI (confidence intervals) are given in the table below. AE = adverse events (syncope, presyncope, VT).

In 26 out of 100 patients (26%) and in 22 (28%) of the patients investigated for orthostatic hypotension (n = 80) the QTc interval was prolonged. While 22 of 26 patients (85%) with QTc prolongation had an adverse event, this was seen in only 32 of 74 patients (43%) with a normal QTc interval (Fig 2). Thus the chance of having an adverse event was enhanced by a factor of 6.6 (95%-CI [2.0;21.7]; p = 0.002) for patients with a QTc prolongation. The chance for syncope was enhanced by a factor of 5.5 (95%-CI [1.9;16.5]; p = 0.002) and for syncope and presyncope by a factor of 3.8 (95%-CI [1.4;10.4]; p = 0.009) for all patients. Although the positive predictive value (PPV) for the occurrence of syncope in patients with a prolonged QTc interval was relatively low (46%), the NPV was considerably high with 88%. Similar values were calculated for syncope and presyncope together with a PPV of 62% and a NPV of 73% (Fig 2). Notably, there was no association between a prolonged QTc interval and the occurrence of VT.

**Fig 3:**
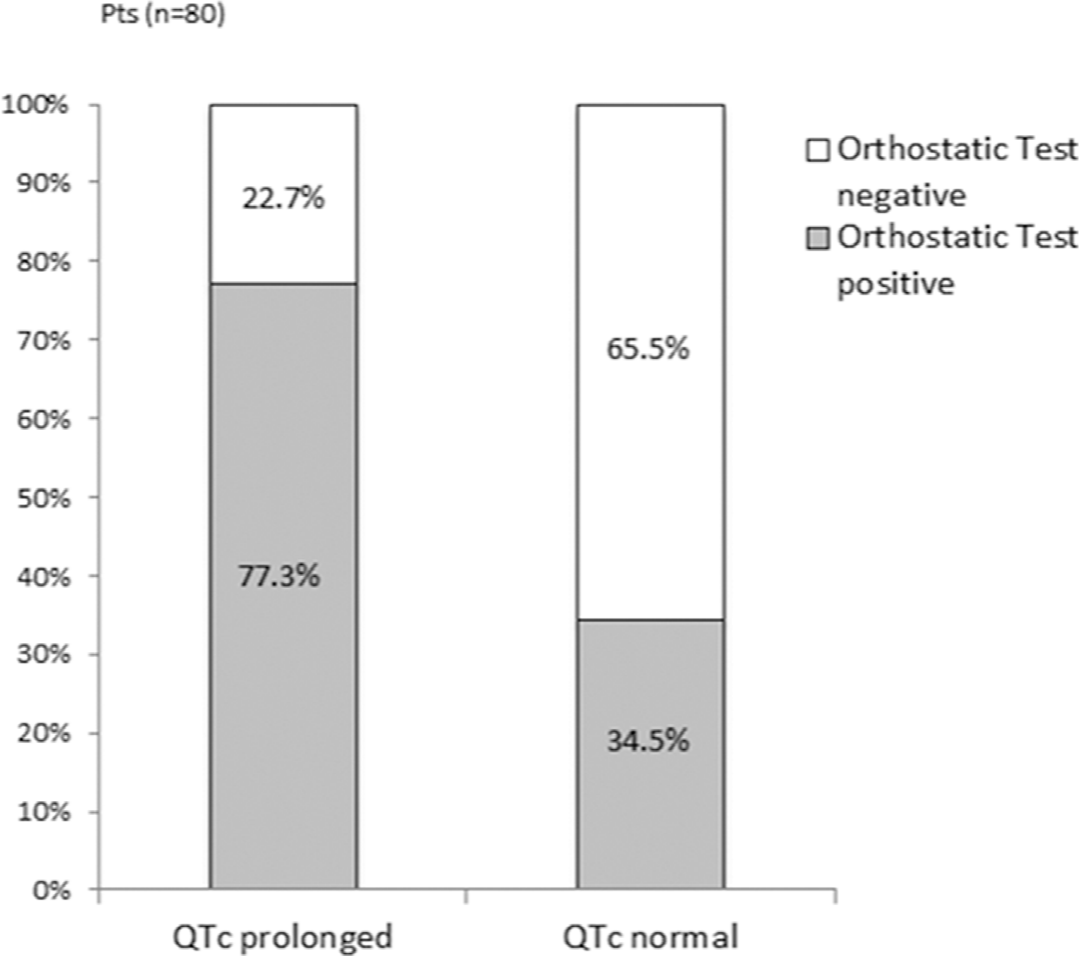
Relationship between orthostatic test result and QTc prolongation. The percentages of patients with a positive and negative orthostatic test result are given for the group with and without QTc prolongation.

**Orthostatic test result and QTc interval as joint predictors for adverse events** Of all patients with QTc prolongation, 77% (n = 17) also showed a positive orthostatic test result, whereas 35% (n = 20) of the patients with a normal QTc had a physiological orthostatic reaction. A total of 25 patients (53%) had either a positive orthostatic test or a prolonged QTc (Fig 3).

While QTc prolongation was a significant predictor for adverse events (Fig 2), no significant impact could be detected when the orthostatic test result was added to the model. The probability for an adverse event with no positive test was 23%, with QTc prolongation 61%, with positive orthostatic test 90% and with both tests positive 94%. The probability for an adverse event with at least one of the two tests was 84%.

## Discussion

These data provide strong evidence that the orthostatic blood pressure test is sensitive and highly specific relating to the occurrence of syncope in HCM patients. They suggest that syncope in HCM patients over the age of 40 years might not be primarily associated with VT, which, in turn, is the putative mechanism for SCD in HCM. In these patients, syncope and presyncope might rather be related to orthostatic hypotension. In line with other recently published data suggesting that asymptomatic VT in older HCM patients fails to predict SCD [12], this observation underlines the currently published guidelines providing a new risk score for HCM in which advanced age is associated with a lower risk score [3].

The orthostatic blood pressure test is designed to assess orthostatic blood pressure and heart rate reactions by observing the physiologic process of rising from a lying position and is therefore predominantly able to objectify orthostatic dysregulation [13–15]. The test is well established in evaluating otherwise unexplained vertigo and cardiac syncope in non-HCM patients [2]. It is very easily and quickly performed, and can be carried out by all patients who are able to stand up unassisted. Furthermore, the test can be performed by a single nurse without support or surveillance of a physician and is therefore quick and cost effective. Abnormal blood pressure response to physical exercise has previously been established to be suitable for identifying HCM patients at risk for SCD [9]. This has also recently become part of the AHA and ESC guidelines for risk stratification of SCD in HCM patients younger than 40 years [3].

Although the overall prevalence of orthostatic hypotension is low [16], it is known to occur in 30–50% of elderly patients with cardiovascular comorbidities and is judged as an independent risk factor for cardiovascular disease, kidney disease, and death [17–19]. In line with other studies using orthostatic blood pressure tests in otherwise healthy individuals [20,21] an age-dependent effect on the prevalence of orthostatic hypotension was also visible in our cohort of HCM patients. This might be explained by a higher intake of antihypertensive medication in older patients, but could also be due to the fact that heart rate response to orthostatic manoeuver is slightly impaired, and arterial compliance and venous system tortuosity increase with advanced age [19,22]. In approximately one third of HCM patients, blood pressure fails to increase during upright exercise [23,24]. Vascular instability has also been suspected to result in hypotension during ordinary daily activity and was previously interpreted as an important cause of syncope in HCM [23]. This can be attributed to an inappropriate vasodilator response in non-exercising vascular beds, leading to an exaggerated fall in systemic vascular resistance. The effect is referred to an enhanced cardiac baroreceptor activity [25] and it has been previously postulated that vascular instability might act as a trigger for syncope and even SCD in patients with an underlying electrophysiologic substrate [23].

In the present study only one positive orthostatic test result was documented among HCM patients younger than 40 years, regardless of comorbidities, the state of the disease and above all, the frequency of VT. Moreover, VT was equally distributed among patients with positive and negative test results. Hence, independent of the patients' age, no correlation between the orthostatic test result and arrhythmogenic events could be revealed.

Compared to normal subjects HCM patients generally seem to have a prolonged QTc interval, which has been attributed to conduction disturbances due to thickened intraventricular walls [26]. Still, hyperreactivity of the adrenergic system and an imbalance in sympathetic and parasympathetic tone have also been described in patients with HCM. Particularly, differences in sympathetic tone have been shown to cause much greater differences in QT intervals in these patients than in healthy controls [27,28]. However, a delay in cardiac repolarisation is known to create an electrophysiological environment that favors the development of life threatening ventricular arrhythmias. Hence, an increased proarrhythmic potential was seen in HCM patients with QT prolongation, independently of the respective maximum wall thickness [29,30]. In addition, prolonged QTc intervals have recently been suggested as an additional risk factor of SCD in HCM [30]. In this study, a prolonged QTc interval was associated with a higher rate of adverse events, although a correlation with the occurrence of VT was not evident. However, patients with a normal QTc interval and a negative orthostatic test result have a very low chance for incurring any adverse event. Furthermore, there are high chances for the occurrence of adverse events in patients with either only QTc prolongation or pathologic orthostatic blood pressure response or both. Nevertheless, taking the orthostatic test into account a prolonged QTc interval did not prove to be of any additional diagnostic value and does not seem to be suitable as an additional test in risk stratification compared to the orthostatic blood pressure test. Thus, the mechanism of adverse events in patients with prolonged QT intervals remains unclear, suggesting that the QTc may be a marker of a more severe phenotype, rather than a cause.

Consistent with previous data [31] NT-proBNP values were above normal range in 81%, and more than ten times increased in 23% of all HCM patients. However, in contrast to others showing a certain dependency of the LVOT gradient and LV hypertrophy on QTc prolongation [29], neither NT-proBNP values, nor other parameters such as cardiac medication, septal wall thickness, or the LVOT gradient had a significant influence on orthostatic test results or QTc intervals.

Taken together, the risk of SCD in HCM patients is increased in all mutation carriers independent of their clinical phenotype, causing individual risk stratification to be challenging. Syncope is valued as one major risk factor in this context. Our data support that the orthostatic blood pressure test proves to be an independent parameter for identification of patients at high risk for syncope and presyncope, providing the most reliable results in patients older than 40 years. However, a correlation with arrhythmogenic events could not be seen. This study demonstrates that orthostatic impairment is a more common cause of syncope and presyncope in HCM than ventricular arrhythmias, with a greater preponderance in older patients, which may explain why syncope in older patients is not as strong a risk factor for SCD than in younger patients. Taking into account that high rates of inappropriate ICD therapies [32] and adverse events during ICD discharges have been previously reported [33], there is a need for better screening parameters to identify HCM patients at high risk for SCD as candidates for primary prevention. In line with the observation that the risk of SCD in HCM patients seems to decrease with proceeding age, syncope in the elderly HCM patient might rather be induced by orthostatic dysregulation than by malignant arrhythmias. We therefore postulate that the orthostatic blood pressure test should be recommended as an additional parameter for further evaluation of syncope in HCM patients older than 40 years.

## Limitations

The chance of detecting VT via 24h-ECG performed once or twice a year was quite low and our population comprised only a relatively small number of patients with an ICD (30%) and documented VT in the device memory, so that some VT might have been missed.

Due to a short observation period and an overall low rate of SCD in this cohort, the prognostic value of the orthostatic test and QTc prolongation on SCD was not investigated. Hence, we can only deduce a very high or low risk for syncope but cannot conclude that this would ultimately lead to a fatal event.
